# Factors associated with sexual and reproductive health behaviour of street-involved young people: findings from a baseline survey in Southwest Nigeria

**DOI:** 10.1186/s12978-020-00937-4

**Published:** 2020-06-11

**Authors:** Atinuke O. Olaleye, Mary O. Obiyan, Morenike O. Folayan

**Affiliations:** 1 Department of Obstetrics and Gynecology, Babcock University, Ilishan, Nigeria; 2 Department of Demography and Social Statistics, Obafemi Awolowo University, Ile-Ife, Nigeria; 3 Department of Child Dental Health, Obafemi Awolowo University, Ile-Ife, Nigeria

**Keywords:** Street youth, Street children, Contraception, Adolescents, Sexual and reproductive health, Nigeria

## Abstract

**Background:**

To achieve the Sustainable Development Goal 3, which is to ensure healthy lives and promote well-being for all persons of all ages, street-involved young people (SIYP) must be assured of universal access to sexual and reproductive healthcare. This study aims to determine the factors associated with age- and sex-specific differences in the sexual and reproductive health (SRH) behaviour of SIYP in southwest Nigeria.

**Methods:**

This was a cross-sectional study that recruited 1505 SIYP aged 10–24 years by use of respondent-driven and time-location sampling. Data were collected through interviewer-administered questionnaires on socioeconomic characteristics; access to SRH information; contraceptive knowledge and use; sexual behavior; and sexual practice. The outcome variables were inconsistent condom use, multiple sexual partners, and transactional sex. Binomial regression analysis models were developed to determine risk indicators for outcome variables.

**Results:**

Although 968 (64.3%) participants were sexually active and 1089 (72.4%) knew about modern contraception, only 300 (31.0%) sexually active respondents used modern contraceptives. Knowledge of modern contraception (AOR: 0.11; 95% C.I: 0.01–0.82, *p* = 0.03) and being employed (AOR: 0.38; 95% C.I: 0.15–0.95, *p* = 0.04) reduced the odds for inconsistent condom use among male SIYPs. For female SIYPs, knowledge of modern contraception reduced the odds for inconsistent condom use (AOR: 0.26; 95% C.I: 0.08–0.90, *p* = 0.03), whereas access to SRH information significantly increased the odds for inconsistent condom use (AOR: 5.06; 95% C.I: 1.67–15.37, *p* = 0.004).

**Conclusion:**

Age- and sex- related factors associated with risky sexual behaviors vary among SIYP. Addressing these differences in the delivery of targeted interventions to reduce sexual health risk of SIYP may be required.

## Plain english summary

Street-involved young people (SIYP) are a vulnerable segment of the population that requires universal access to sexual and reproductive healthcare. This study was conducted among 1505 SIYP in southwest Nigeria to determine the factors associated with age- and sex- specific differences in their sexual and reproductive health (SRH) behaviour. Using interviewer-administered questionnaires, information was obtained on the socioeconomic characteristics, access to SRH information, contraceptive knowledge and use, and sexual behavior and sexual practice of the respondents. Sexual-risk behavior included inconsistent condom use, multiple sexual partners, and transactional sex. Findings from the study revealed that despite a relatively high awareness of modern contraception, fewer than a third of sexually active SIYP use modern contraceptives. There were also age and sex differences in the sexual risk behaviors of SIYP. These findings imply that SIYP need age- and sex- segmented targeted interventions to address their SRH needs.

## Background

Access of adolescents and young persons to culturally sensitive messages on SRH is still inadequate; there is limited context-specific evidence to facilitate the development of culturally appropriate information for the diverse populations of young people [1, 2]. To develop strategic actions for the non-homogenous population of young persons, stratification based on their social context and vulnerability is necessary [3, 4]. Population stratification facilitates the design and implementation of programs that enhance equitable access to services. A vulnerable segment of street-involved young people (SIYP) are those who live “on the street” (work on the streets but return home at night or maintain contact with families) and those who live “of the street” (those who never return home or have lost contact with families) [5, 6]. A street child is: “*Any girl or boy who has not reached adulthood, for whom the street in the widest sense of the word, including unoccupied dwellings, wasteland, and so on, has become his or her habitual abode and/or source of livelihood, and who is inadequately protected, directed, and supervised by responsible adults*” [7]. Several million children and youths are street-connected, with the largest burden in low and middle-income countries [8–10].

SIYP have limited understanding of and access to information on SRH, making it challenging for them to make healthy choices, which increases their prospect for having risky sexual behavior, such as unprotected sexual intercourse, early age of sexual debut, multiple sexual partners, and transactional sex [11, 12].

The psychosocial framework for understanding adolescent risk behavior described by Jessor [13] helps our understanding of the complex interrelationship of the risk (and protective) factors that influence risk behavior and consequent health or life outcomes of adolescents. Risky sexual behavior of young people has been described at the personal, family, peer, school and community levels [14]. Personal factors such as age, level of education, sexual knowledge and sources of sex information have been implicated [15, 16]. Family structure and relationships, peer influence, religious beliefs, economic constraints, and social changes in the community also influence adolescents’ sexual decision-making [14, 17]. Poor access of adolescents and young persons to SRH services because of social stigmatization and marginalization makes it challenging for SIYP to access SRH-related treatment, with attendant complications [11, 18]. These SRH-related risk factors fit into four of the five domains described by Jessor [13]: the social environment, the perceived environment, personality, other behavior, and biology/genetics.

SIYP are often hidden and thus neglected in most public health interventions [19]. They therefore bear a substantial burden of SRH needs, including unintended pregnancy and unsafe abortion. One of the goals of Sustainable Development Goal 3 is that of countries ensuring universal access to SRH-care services by 2030, including access to family planning information and education, and the integration of reproductive health into national strategies and programs [20]. To meet this goal, it is important to understand how the interplay of social, economic, and cultural factors influence the sexual behaviors of young people, including SIYP. This study addresses one of these gaps: the factors associated with age- and sex-specific differences in SRH behaviour of SIYP who reside in an industrialized versus a less industrialized town in southwest Nigeria. Industrialization has contributed to influx of people, especially young people, into cities in a bid to earn a better living. However, congestion, limited opportunities, and scarce economic resources drive many young people into the streets. Our recruitment of SIYP was therefore from industrialized and less industrialized rather than urban and rural Nigeria. We hypothesize that there will be age- and sex-specific differences in SRH behaviour of SIYP by residential areas.

## Methods

### Study design, population and study sites

This is a cross-sectional study conducted to determine the SRH needs of SIYPs in two states in southwestern Nigeria. The two States – Lagos and Osun -- were selected based on their level of industrialization. Lagos is an industrialized cosmopolitan state, while Osun is less industrialized. Data were collected in January to February, 2019.

The study participants were male and female adolescents aged 10 to 24 years, living ‘on’ and ‘of’ the street. At each study location, places where SIYPs congregate in large numbers, such as major streets, market places, and motor-parks, were identified through mapping conducted by the research team and officials of the State Ministry of Health. These locations were grouped as clusters. The clusters in Lagos State were Bariga and Ajah, and those for Osun State were Oke-Baale, Olaiya and Sabo.

### Sample size

The determination of sample size for this study was guided by Turner [21], who recommended estimates to derive sample size for surveys on orphaned and vulnerable children (OVC) in homeless situations. Because of unavailability of data to generate a prevalence rate of OVC in the proposed study environment, the suggested minimum sample size of 800 to 1000 was adopted, and 1505 street-involved young people were enrolled in this study.

### Study recruitment procedure

After community engagement, the study participants were recruited by use of respondent-driven sampling and time-location sampling methods [22, 23]. The respondent-driven sampling method developed by Heckathorn [24] is a sampling process whereby participants recruit their peers in hard-to-reach populations [25]. In this study, the first 10 seeds recruited through the respondent-driven sampling were given a labelled tag with generated identity numbers. After their enrollment, the seeds were given two additional tags to recruit friends/peers. Each referred respondent was checked for eligibility, enrolled, and interviewed once the eligibility criteria were met. The eligibility criteria were age 10–24 years, living ‘on’ or ‘of’ the street in Lagos or Osun State, and stable mental status.

Recruitment of study participants through respondent-driven sampling was slow, as there were boundaries within street groups and low density of social networks. Thus, the research team adopted time-location sampling to recruit the target population at specific times, days, and venues where SIYP gather [26]. To limit selection bias with this method, we selected venue-day-time options with likely large turnouts of SIYP for the recruitment of participants.

Through the respondent-driven sampling, 34 seeds were recruited, and 465 coupons were given out over six recruitment waves within 5 days; only 120 respondents were recruited and interviewed through this method. In contrast, the time-location sampling method resulted in 1800 coupons given and 1385 respondents recruited and interviewed (See Supplementary File 1 for study recruitment characteristics).

The questionnaire was administered in a place that the respondent identified to be most comfortable for responding to questions. Data were collected by the field worker electronically with REDcap - a secure web application used to build and manage online surveys/databases. The REDcap app was installed on tablets for offline use, and all data collected were uploaded to the secured website at the end of each day.

### Study instrument

The research instrument was adapted from the “Illustrative Questionnaire for Interview-Surveys with Young People,” designed by Cleland for the World Health Organisation [27]. The content of the questionnaire was revised by two experts in SRH to address the study objectives and fit within the Nigerian cultural context. The revised questionnaire was pre-tested with 20 selected SIYPs at two locations besides the study sites (Ile-Ife and Ibadan) to ascertain the clarity and conciseness of the questions. The tool was revised for language and procedural clarity, then translated into the local dialect (Yoruba) for respondents who do not understand English language. The questions were closed-ended, making data entry and analysis possible in English. Field workers who speak the other national languages - Ibo and Hausa – had access to interpreted key concepts in the questionnaire in line with the methodology used for national health surveys conducted in Nigeria [28].

### Measurement of variables

The explanatory variable in this study was ‘knowledge of SRH’ measured by (i) knowledge of modern contraceptives and (ii) access to SRH information. A knowledge of modern contraceptives was deduced from responses to a question asked from respondents - “do you know of any of these methods which men and women can use to prevent pregnancy?” There were seven response options: (i) injection, (ii) condom, (iii) emergency contraception, (iv) traditional method, (v) withdrawal method, (vi) safe period and (vii) periodic abstinence. Options i-iii were recoded as ‘1’, implying knowledge of modern method of contraception, and ‘0’, no knowledge of modern contraception. Further, respondents were asked if they ‘ever attended/or were given a talk on SRH’; a positive response was assigned “1”, and otherwise “0”. The question was limited to information on SRH learned through talks since most SIYPs likely have restricted access to other sources of information, such as electronic media and social media. The respondents were also asked to state the contraceptive method they used at last sexual activity by self or partner.

The variables adjusted for in this study were selected background characteristics of respondents: age [10–14, 15–19, 20–24 years], level of education [none, primary, secondary], and employment status [not working, working]. The outcome variable was ‘sexual risk behavior’ proxied by three variables: (i) inconsistent use of condom, (ii) multiple sexual partners; and (iii) transactional sex. Both male and female respondents who were sexually active were asked if they used condom at the last sexual activity. An affirmative answer was assigned “1”, and a negative answer was assigned “0”. Respondents were also asked the number of sex partners they currently have. Those who responded that they had one were assigned “1”, while those who had more than one sex partner were assigned “0”. Two questions were asked to probe about transactional sex: “have you ever paid or exchanged gift for sex?” and “have you ever been paid or receive gift in exchange for sex?” An affirmative answer to either of the questions was assigned “1”, and a negative answer was assigned “0”.

### Analysis

Data analysis was conducted with Stata SE 15.1 (Stata Corporation, College Station, Texas). The univariate analysis was conducted to determine the percentage distribution of participants by age in grouped years, sex, educational level, employment status, knowledge of modern contraception, and access to SRH information. Bivariate analysis was conducted to test associations between the explanatory and outcome variables by use of Pearson chi-square test. The inferential analysis was conducted with logistic regression to determine the risk indicators for the outcome variables by sex. Two models guided the regression analysis: the first model regressed each of the outcomes against the explanatory variables, while the second model adjusted for confounders (age, education level, and work status).

Based on the Pearson chi-square significant association test, we set the *p*-value cut-off point at 0.20 (*p* < 0.20) for the inclusion of confounders in the regression model. The Hosmer-Lemeshow goodness-of-fit test was conducted to ascertain that all study variables fulfilled the underlying assumption of a univariate regression. Statistical significance was considered at *p*-value less or equal to 0.05.

## Results

### Background characteristics of study participants

Missing data was less than 1%. The distribution of the respondents by age, level of education, and work status is shown in Table 1. There were more males (57.6%) than females (42.4%), with a mean (standard deviation) age of 17.9 (3.89) for male respondents and 17.7 (3.74) for the female respondents. Most of the respondents were in the 15–19-year age group (41.3%), had no formal education (50.8%), and were unemployed at the time of the interview (57.2%).

**Table 1:** Individual Characteristics and Knowledge of Sexual and Reproductive Health of SIYPs by Sex and Age (*N* = 1505)

Individual Characteristics	Male (*N* = 867) n (%)	Female (*N* = 638) n (%)	X^2^; *p* value	Total (*N* = 1505) n (%)
Variables				
Age Group	Mean Age – 17.93	Mean Age – 17.65		
10–14	185 (21.3%)	138 (21.6%)	X^2^ = 9.33 p = 0.01	323 (21.5%)
15–19	333 (38.4%)	289 (45.3%)		622 (41.3%)
20–24	349 (40.3%)	211 (33.1%)		560 (37.2%)
Level of Education				
None	464 (53.5%)	300 (47.0%)	X^2^ = 6.25 *p* = 0.044	764 (50.8%)
Primary	182 (21.0%)	150 (23.5%)		332 (22.1%)
Secondary	221 (25.5%)	188 (29.5%)		409 (27.1%)
Employment Status				
Not working	449 (51.8%)	412 (64.6%)	X^2^ = 24.56 *p* < 0.001	861 (57.2%)
Working	418 (48.2%)	226 (35.4%)		644 (42.8%)
Knowledge of modern contraceptives				
No	262 (30.2%)	154 (24.1%)	X^2^ = 6.80 *p* < 0.01	416 (27.6%)
Yes	605 (69.8%)	484 (75.9%)		1089 (72.4%)
Access to SRH information				
No	764 (88.1%)	500 (78.4%)	X^2^ = 25.98 *p* < 0.001	1264 (84.0%)
Yes	103 (11.9%)	138 (21.6%)		241 (16.0%)
Ever had sexual relationship				
No	319 (36.8%)	218 (34.2%)	X^2^ = 1.10 *p* = 0.29	537 (35.7%)
Yes	548 (63.2%)	420 (65.8%)		968 (64.3%)
Knowledge of Sexual and Reproductive Health by Age				
Variables	**10–14 (*N*** **= 323)** n (%)	**15–19 (*N*** **= 622)** n (%)	**20–24 (*N*** **= 560)** n (%)	**X**^**2**^**; p value**
Knowledge of modern contraceptives				
No (416)	131 (40.6%)	140 (22.5%)	145 (25.9%)	X^2^ = 35.99 p < 0.001
Yes (1089)	192 (59.4%)	482 (77.5%)	415 (74.1%)	
Access to SRH information				
No (1264)	311 (96.3%)	518 (83.4%)	435 (77.7%)	X^2^ = 50.88 p < 0.001
Yes (241)	12 (3.7%)	104 (16.7%)	125 (22.3%)	

### Knowledge of sexual and reproductive health

Table 1 also shows the age and sex distribution of respondents by their knowledge about SRH. Most (72.4%) of the respondents knew about modern methods of contraception, although the majority (84.0%) had no access to SRH information. Those who had access to information (16%) received it through formal talks or at health facilities. More female than male respondents knew about modern contraceptive methods (75.9% vs 69.8%; *p* = 0.01) and had access to SRH information (21.6% vs 11.9%; *p* < 0.001). Also, significantly more SIYPs who were 10–14 years old than those who were 15–19 years old or 20–24 years old did not know about modern contraception (*p* < 0.001) and had no access to SRH information (*p* < 0.001).

### Sexual and reproductive health behavior of sexually active SIYPs

Table 2 highlights the sex distribution of sexually active SIYPs by their sexual behavior. Of the 1505 participants recruited, 968 (64.3%) admitted to being sexually active. Of the sexually active SIYP, 300 (31.0%) had ever used any form of modern contraception. The main form of contraception used was the condom. The majority (93.3%) of sexually active SIYP used the condom inconsistently; more than half (56.0%) had multiple sexual partners; and 221 (22.8%) engaged in transactional sex. More females than males reported ever using modern contraception (*p* = 0.27); used the condom inconsistently (*p* < 0.002); had multiple sexual partners (*p* < 0.01); and engaged in transactional sex (*p* < 0.03).

**Table 2:** Sexual health practices among sexually active SIYP by Sex in South-West, Nigeria (*N* = 968)

Variables	Male (*N* = 548) n (%)	Female (*N* = 420) N (%)	X^2^; *p* value	Total (*N* = 968) (%)
Contraceptive Method used at last sexual activity^a^				
Condom	130 (23.7%)	106 (25.2%)	X^2^ = 1.99 *p* = 0.16	236 (24.4%)
Pills	12 (2.2%)	52 (12.4%)		64 (6.6%)
Withdrawal method	15 (2.7%)	19 (4.5%)		34 (3.5%)
Safe Period	6 (1.1%)	9 (2.1%)		15 (1.6%)
Traditional	1 (0.2%)	10 (2.4%)		11 (1.1%)
Multiple Partners				
one sexual partner	222 (40.5%)	204 (48.6%)	X^2^ = 6.27 *p* < 0.01	426 (28.3%)
more than one sexual partner	326 (59.5%)	216 (51.4%)		542 (56.0%)
Transactional Sex				
No	409 (74.6%)	338 (80.5%)	X^2^ = 4.60 *p* = 0.03	747 (77.2%)
Yes	139 (25.4%)	82 (19.5%)		221 (22.8%)
Inconsistent use of condom				
No	25 (4.6%)	40 (9.5%)	X^2^ = 9.34 *p* < 0.002	65 (6.7%)
Yes	523 (95.4%)	380 (90.5%)		903 (93.3%)

^a^contraceptive method used at last sexual activity by self or partner

### Sexual behavior of SIYP by sex and age

Table 3 highlights the sex differences in the sexual risk profile of sexually active SIYPs, and Table 4 highlights the age differences. In inconsistent use of condom, there were significantly more male than female SIYP who were 20–24 years old (*p* < 0.01); more females than males who were 15–19 years old (*p* < 0.01); and more females than males who knew about modern contraception (*p* < 0.001). Also, more 20–24-year-old males and 15–19-year-old females (*p* < 0.01) had multiple sex partners.

**Table 3:** Background characteristics, SRH knowledge and sexual risk profile of sexually active SIYP by sex (*N* = 968)

Variables	Inconsistent Use of Condom (*N* = 903)			Multiple Partners (*N* = 542)			Transactional Sex (*N* = 221)		
	Male (***N*** = 523) n (%)	Female (***N*** = 380) n (%)	Χ^**2**^ (***p***-value)	Male (***N*** = 326) n (%)	Female (***N*** = 216) n (%)	Χ^**2**^ (***p***-value)	Male (***N*** = 139) n (%)	Female ***N*** = (82) n (%)	Χ^**2**^ (***p***-value)
Age Group									
10–14 years	98 (18. 7%)	76 (20.0%)	**Χ**^**2**^ = 8.49 *p* = 0.01	57 (17.5%)	46 (21.3%	**Χ**^**2**^ = 4.18 *p* = 0.12	15 (10.8%)	12 (14.6%)	**Χ**^**2**^ = 7.56 *p* = 0.02
15–19 years	194 (37.1%)	179 (47.1%)		120 (36.8%)	87 (40.3%)		58 (41.7%)	38 (46.3%)	
20–24 years	231 (44.2%)	125 (32.9%)		149 (45.7%)	83 (38.4%)		66 (47.5%)	32 (39.0%)	
Level of Education									
None	275 (52.6%)	170 (44.8%)	**Χ**^**2**^ = 7.60 *p* = 0.02	164 (50.3%)	106 (49.1%)	**Χ**^**2**^ = 6.17 *p* = 0.05	53 (38.1%)	31 (37.8%)	**Χ**^**2**^ = 12.53 *p* = 0.002
Primary	117 (22.4%)	95 (25.0%)		73 (22.4%)	37 (17.1%)		36 (25.9%)	25 (30.5%)	
Secondary	131 (25.1%}	115 (30.3%)		89 (27.3%)	73 (33.8%)		50 (36.0%)	26 (31.7%)	
Employment status									
Not working	270 (51.6%)	241 (63.6%)	**Χ**^**2**^ = 3.56 *p* = 0.06	174 (53.4%)	130 (60.5%)	**Χ**^**2**^ = 0.06 *p* = 0.81	78 (56.1%)	54 (67.5%)	**Χ**^**2**^ = 2.24 *p* = 0.13
Working	253 (48.4%)	138 (36.4%)		152 (46.6%)	85 (39.5%)		61 (43.9%)	26 (32.5%)	
Knowledge of modern contraceptives									
No	155 (29.6%)	83 (21.8%)	**Χ**^**2**^ = 13.20 *p* < 0.001	88 (27.0%)	51 (23.6%)	**Χ**^**2**^ = 0.27 *p* = 0.60	17 (12.2%)	10 (12.2%)	**Χ**^**2**^ = 24.96 *p* < 0.001
Yes	368 (70.4%)	297 (78.2%)		238 (73.0%)	165 (76.4%)		122 (87.8%)	72 (87.8%)	
Access to SRH information									
No	460 (88.0%)	287 (75.5%)	**Χ**^**2**^ = 0.15 *p* = 0.70	284 (87.1%)	163 (75.5%)	**Χ**^**2**^ = 0.13 *p* = 0.72	114 (82.0%)	59 (72.0%)	**Χ**^**2**^ = 4.21 *p* = 0.04
Yes	63 (12.0%)	93 (24.5%)		42 (12.9%)	53 (24.5%)		25 (18.0%)	23 (28.0%)

**Table 4:** Background characteristics, SRH knowledge and sexual risk profile of sexually active SIYPs by age group (*N* = 968)

Variables	Inconsistent Use of Condom (*n* = 903)			Multiple Partners (*n* = 542)			Transactional Sex (*n* = 221)		
	10–14 years (*N* = 174) n (%)	15–19 years (*N* = 373) n (%)	20–24 years (*N* = 356) n (%)	10–14 years (*N* = 103) n (%)	15–19 years (*N* = 207) n (%)	20–24 years (*N* = 232) n (%)	10–14 years (*N* = 27) n (%)	15–19 years (*N* = 96) n (%)	20–24 years (*N* = 98) n (%)
Gender									
Male	98 (56.3%)	194 (52.0%)	231 (64.9%)	57 (55.3%)	120 (58.0%)	149 (64.2%)	15 (55.6%)	58 (60.4%)	66 (67.3%)
Female	76 (43.7%)	179 (48.0%)	125 (35.1%)	46 (44.7%)	87 (42.0%)	83 (35.8%)	12 (44.4%)	38 (39.6%)	32 (32.7%)
*X*^*2*^*; p value*	9.34; *p* = 0.002			6.27; *p* < 0.01			4.60; *p* = 0.03		
Level of Education									
None	119 (68.4%)	176 (47.2%)	150 (42.1%)	74 (71.8%)	104 (50.2%)	92 (39.7%)	18 (66.7%)	38 (39.6%)	28 (28.6%)
Primary	47 (27.0%)	84 (22.5%)	81 (22.8%)	25 (24.3%)	37 (17.9%)	48 (20.7%)	8 (29.6%)	25 (26.0%)	28 (28.6%)
Secondary	8 (4.6%)	113 (30.3%)	125 (31.1%)	4 (3.9%)	66 (31.9)	92 (39.7%)	1 (3.7%)	33 (34.4%)	42 (42.9%)
*X*^*2*^*; p value*	3.02; *p* = 0.08			0.69; *p* = 0.41			5.93; *p* = 0.02		
Employment Status									
Not working	141 (81.0%)	222 (59.7%)	148 (41.6%)	88 (85.4%)	115 (55.8%)	101 (43.5%)	19 (70.4%)	63 (65.6%)	50 (52.1%)
Working	33 (19.0%)	150 (40.3%)	208 (58.4%)	15 (14.6%)	91 (44.2%)	131 (56.5%)	8 (29.6%)	33 (34.4%)	46 (47.9%)
*X*^*2*^*; p value*	3.56; *p* = 0.06			0.06; *p* = 0.81			2.24; *p* = 0.13		
Knowledge of modern contraceptives									
No	74 (42.5%)	71 (19.0%)	93 (26.1%)	48 (46.6%)	138 (8.4%)	53 (22.8%)	8 (29.6%)	10 (10.4%)	9 (9.2%)
Yes	100 (57.5%)	302 (81.0%)	263 (73.9%)	55 (53.4%)	169 (81.6%)	179 (77.2%)	19 (70.4%)	86 (89.6%)	89 (90.8%)
*X*^*2*^*; p value*	13.20; *p* < 0.001			0.27; *p* = 0.60			24.96; *p* < 0.001		
Access to SRH information									
No	170 (97.7%)	304 (81.5%)	273 (76.7%)	99 (96.1%)	164 (79.2%)	184 (79.3%)	25 (92.6%)	77 (80.2%)	71 (72.5%)
Yes	4 (2.3%)	69 (18.5%)	83 (23.3%)	4 (3.9%)	43 (20.8%)	48 (20.7%)	2 (7.4%)	19 (19.8%)	27 (27.5%)
*X*^*2*^*; p value*	0.15; *p* = 0.70			0.12; *p* = 0.72			4.21; *p* = 0.04		

Further, the study shows that more male SIYPs who were 20–24 years old and more females who were 15–19 years old (*p* = 0.02); more males who can read and write (*p* = 0.02); more 10–14-year-old adolescents who cannot read and write (*p* = 0.02); and more 10–14-year-old adolescents who had knowledge of modern contraception (*p* < 0.001) engaged in transactional sex.

### Indicators for sexual risk behavior for male and female SIYP

The outcomes of the logistic regression determining the risk indicators for the three sexual risk behaviors of male SIYPs (Table 5) and female SIYPs (Table 6) are given. Among male SIYPs, knowledge about modern contraception significantly reduced the odds of inconsistent use of condom when compared with no knowledge (OR: 0.10; 95% CI: 0.01–0.77; AOR: 0.11; 95% CI: 0.01–0.82).

**Table 5:** Logistic regression of sexual-risk behavior on knowledge and SRH information access among sexually-active Male SIYP (*N* = 548)

Variables	Model I: (Crude Odds Ratio)						Model II (Adjusted Odds Ratio)					
	Inconsistent Use of Condom		Multiple Partners		Transactional Sex		Inconsistent Use of Condom		Multiple Partners		Transactional Sex	
Knowledge of modern contraceptives	OR	C I at 95%	OR	C I at 95%	OR	C I at 95%	AOR	C I at 95%	AOR	C I at 95%	AOR	C I at 95%
No	1.00	–	1.00	–	1.00	–	1.00	–	1.00	–	1.00	–
Yes	0.10	0.01–0.77 *	1.18	0.80–1.72	3.49	2.01–6.07 *	0.11	0.01–0.82 *	1.15	0.77–1.70	3.01	1.71–5.29 *
Access to SRH information												
No	1.00	–	1.00	–	1.00	–	1.00	–	1.00	–	1.00	–
Yes	0.75	0.27–2.08	1.12	0.65–1.91	1.50	0.87–2.59	1.20	0.38–3.84	1.03	0.58–1.81	1.20	0.67–2.16
Age Group												
10–14 years							1.00	–	1.00	–	1.00	–
15–19 years							0.69	0.14–3.42	1.00	0.61–1.66	1.77	0.91–3.44
20–24 years							0.56	0.12–2.67	1.09	0.67–1.78	1.83	0.94–3.54
Level of Education												
None							1.00	–	1.00	–	1.00	–
Primary							0.63	0.22–1.79	1.02	0.66–1.57	1.43	0.86–2.38
Secondary							0.73	0.27–2.02	1.21	0.77–1.92	1.75	1.06–2.89*
Employment Status												
Not working							1.00	–	1.00	–	1.00	–
Working							0.38	0.15–0.95 *			0.64	0.43–0.98*

*p < 0.05

**Table 6:** Logistic regression of sexual-risk behavior on knowledge and SRH information access among sexually-active female SIYP (*N* = 420)

Variables	Model I: (Crude Odds Ratio)						Model II (Adjusted Odds Ratio)					
	Inconsistent Use of Condom		Multiple Partners		Transactional Sex		Inconsistent Use of Condom		Multiple Partners		Transactional Sex	
Knowledge of modern contraceptives	OR	C I at 95%	OR	C I at 95%	OR	C I at 95%	AOR	C I at 95%	AOR	C I at 95%	AOR	C I at 95%
No	1.00	–	1.00	–	1.00	–	1.00	–	1.00	–	1.00	–
Yes	0.26	0.08–0.86 *	0.65	0.40–1.05	2.01	0.98–4.12	0.27	0.08–0.92*	0.79	0.47–1.31	1.77	0.85–3.72
Access to SRH information												
No	1.00	–	1.00	–	1.00	–	1.00	–	1.00	–	1.00	–
Yes	2.16	0.87–5.34	1.21	0.76–1.91	1.21	0.70–2.10	5.06	1.67–15.30*	1.17	0.71–1.90	1.11	0.61–2.02
Age Group												
10–14 years							1.00	–	1.00	–	1.00	–
15–19 years							0.63	0.17–2.34	0.62	0.35–1.08	1.21	0.57–2.57
20–24 years							0.32	0.08–1.26	0.97	0.53–1.78	1.43	0.63–3.26
Level of education												
None							1.00	–	1.00	–	1.00	–
Primary							1.41	0.51–3.90	0.44	0.26–0.74*	1.64	0.88–3.04
Secondary							0.48	0.21–1.08	0.88	0.53–1.41	1.18	0.63–2.24
Employment Status												
Not working							1.00	–	1.00	–	1.00	–
Working							0.96	0.45–2.03	–	–	0.65	0.37–1.14

*p < 0.05

For the male respondents, being employed reduced the odds of inconsistent use of condom (AOR: 0.38; 95% CI: 0.15–0.95) and engaging in transactional sex (AOR: 0.64; 95% CI: 0.43–0.98) compared with being unemployed. Factors associated with increased odds for sexual risk behavior were having knowledge of modern contraception and a secondary education. Male SIYPs that had knowledge of modern contraception (AOR: 3.01; 95% C.I: 1.71–5.29) and secondary school education (AOR: 1.75; 95% C.I: 1.06–2.89) had increased odds of engaging in transactional sex compared with those with no contraception knowledge and no education, respectively.

Also shown in Table 6, the knowledge about modern contraception reduced the odds for inconsistent condom use when compared with having no such knowledge (OR: 0.26; 95% C.I: 0.08–0.86; AOR: 0.27; 95% C.I: 0.08–0.92) for female respondents. Having primary education reduced the odds of having multiple sexual partners compared with having no education (AOR: 0.44; 95% C.I: 0.26–0.74, *p* = 0.002). A factor associated with increased odds for sexual risk behavior was access to SRH information; female SIYPs who had access to SRH information were five times more likely to use condom inconsistently than those who had no access to SRH information (AOR: 5.06; 95% CI: 1.67–15.30).

## Discussion

The study identified factors associated with age- and sex-specific differences in SRH behaviour of SIYP residing in an industrialized and a less industrialized town in Nigeria. The age- and sex- related SRH risk indicators among SIYPs differed. Among female SIYPs, the older the age group, the less likely was inconsistent condom use and having multiple sexual partners. Among male SIYPs the older the age group, the less likely was inconsistent condom use. While knowledge about modern contraception and education level were risk indicators for male SIYPs to engage in transactional sex, they were not indicators for this activity among female SIYPs. Access to SRH information was a risk indicator for inconsistent condom use by female SIYPs but not for male SIYP. These findings did not differ by industrialized and less industrialized residence.

One strength of this study is its large sample of SIYP, which makes possible a robust analysis. The study also included participants from various cluster areas in each state and from two states with different economic profiles, thereby reducing the risk for non-representation of study participants.

The study highlights the factors predicting age and sex differences in SRH behaviour among SIYPs in southwest Nigeria. First, the study highlights a high unmet need for contraception among SIYPs. The proportion of sexually active female SIYP not using any form of contraception was higher than the 48.4% national estimate of unmet contraception need among sexually active unmarried women [29], and it is higher than the 40.5% reported estimate for 10–24-year-old persons living in households in Sierra Leone [30]. The unmet need for contraception among adolescents in developing countries is high -- more than 40% in most countries in sub-Saharan Africa [31] -- with variations in sex, marital status, age, education and environment [32, 33]. This high unmet need for contraception – a rate higher than the national average - indicates that attention needs to be paid to this large population of adolescents. The 2019 draft national adolescent health policy [34] recognizes for the first time the SRH needs of street-involved young people. However, whether this policy can be translated into programs and interventions for this population once the policy is ratified, remains to be seen.

Second, we found that the contraception option used by SIYP is mostly limited to condoms. Similar observations had been made in an earlier study, and the reason adduced is that condoms are relatively inexpensive and accessible [35]. However, the large proportion of sexually active SIYPs who do not use condom consistently reduces its usefulness in the prevention of unintended pregnancy. As SIYP are already at increased vulnerability to SRH problems, the occurrence of unintended pregnancy likely leads to unsafe abortions, with its attendant adverse consequences. It is therefore essential to vigorously promote the use of more effective, user-independent long-acting reversible contraception (LARC), such as intrauterine devices and implants, in sexually active SIYP who wish to delay childbearing [36].

However, LARC requires a visit to the health facility for insertion and removal. The low utilization of clinics for uptake and use of contraception by adolescents and young persons in Nigeria [37–39] will likely be a barrier for LARC access by SIYP. Poor attention paid to the SRH needs of SIYP will further limit their use of healthcare services for their SRH needs. Where young persons have access to clinics for SRH services, the discordance between the national policy and hospital family planning protocols on LARC further reduces the chances of SIYP accessing LARC. In Nigeria, although the full range of contraceptive services is available, contraception counseling for young people is often limited to barrier methods, pills, and emergency contraception [21]. Efforts are needed to promote not only access of adolescents and young persons to SRH services, but also access of SIYP especially. This effort will need creativity on the part of governments and SRH programmers to promote community rather than facility-based access of SIYP to SRH services. Adolescents in general have difficulty accessing facilities services due to the requirement for parental consent for those less than 18 years, and the stigma associated with unmarried adolescents accessing contraception [40–42]. SIYP may find access even more challenging. Community youth-friendly services will provide healthcare providers the opportunity to screen and counsel SIYP on SRH issues to promote making healthy choices [43]. However, while LARC may address the challenges associated with unwanted pregnancy, it will not address the risk of sexually transmitted infection. SIYP should, therefore, be educated on the need to use condoms consistently to prevent sexually transmitted infection, even when using LARC.

Third, this study identified significant age and sex differences in the sexual risk behavior of SIYP – more females use condom inconsistently, while more males engage in transactional sex. Also, more younger than older adolescent SIYPs engage in risky sexual behavior. This information is important for the design of targeted and segmented interventions for SIYP. Such interventions will best be conducted by adapting SRH risk-reduction messages for different age- and sex- segmented populations [44–46].

Despite the importance of the study findings, there are limitations, First, the fewer positive responses on some variables resulted in wide confidence intervals. Second, the face-to-face interviews increased the risk for social desirability-response bias [47]. Third, the study was conducted in only two of the 36 + 1 tates in Nigeria. There are geographical and cultural variations in the sexual and reproductive lifestyles of Nigerians among the States, so the study findings may not be representative of Nigeria, although likely they are representative of states in the Southwestern part of the country. Fourth, the study is cross-sectional, so we cannot determine a cause-effect relationship between risk factors and SRH behaviours of SIYP in the study populations. Finally, although the Nigeria population is highly religious, we did not measure the possible moderating effect of religiosity on our findings, which is a defect because religiosity moderates SRH behaviour [48, 49].

## Conclusion

Despite the study’s limitations, it reveals that age and sex differences exist in the sexual risk behaviors of SIYP in southwest Nigeria, independent of the area of SIYPs’ residence. Also, there is a high unmet need for contraception and inconsistent condom use among this population. SIYP need targeted interventions for age- and sex- stratified populations to implement programs that address their SRH needs. Qualitative studies can also help further explore the reasons for the observations in this study.

## Supplementary information

**Additional file 1: Supplementary File 1.**. Recruitment Characteristics for Street-Involved Young People in two urban communities, South-West, Nigeria (Jan – Feb 2019)

## Data Availability

The datasets analysed during the current study are available from the corresponding author on reasonable request.

## Abbreviations

AOR: Adjusted odds ratio
CI: Confidence interval
OR: Odds ratio
OVC: Orphaned and vulnerable children
SIYP: Street-involved young people
SRH: Sexual and Reproductive Health
