# Supplementary material for: Factors associated with sexual and reproductive health behaviour of street-involved young people: findings from a baseline survey in Southwest Nigeria
Source: Reprod Health. 2020 Jun 11;17:94. doi: 10.1186/s12978-020-00937-4 (PMC7291518; doi:10.1186/s12978-020-00937-4)
Supplement: Supplementary file 1 — Additional file 1: Supplementary File 1.. Recruitment Characteristics for Street-Involved Young People in two urban communities, South-West, Nigeria (Jan – Feb 2019) [file 12978_2020_937_MOESM1_ESM.docx]

Supplementary File 1: Recruitment Characteristics for Street-Involved Young People in two urban communities, South-West, Nigeria (Jan – Feb 2019)

| Recruitment Method | S/N | Study Area | Lagos | | Osun | Total |
| --- | --- | --- | --- | --- | --- | --- |
|  |  |  | Bariga | Ajah | Osogbo |  |
|  | A | Total Recruitment Time (days) | 5 days | 4 days | 4 days | 13 |
| 1. Respondent Driven Sampling (RDS) | b. | Total number of seeds | 10 | 10 | 14 | 34 |
|  | c. | Total number of coupons given | 191 | 132 | 142 | 465 |
|  | d. | Total number returned coupons, eligible and recruited | 48 | 39 | 33 | 120 |
|  | e. | Duplicate participants dropped | 3 | 3 | 0 | 6 |
|  | f. | Number of recruits by seed (range) | 1-5 | 1-5 | 1-3 | - |
|  | g. | Number of recruitment waves (range) | 1-6 | 1-6 | 1-3 | - |
| 2. Time-Location Sampling | h. | Total Recruitment Time (days) | 7 days | 6 days | 6 days | 19 |
|  | i. | Total number of coupons given | 600 | 500 | 700 | 1800 |
|  | j. | Eligible and recruited respondents | 458 | 338 | 589 | 1,385 |
| Total Respondents Recruited | (sum d and j) | | 506 | 377 | 622 | 1,505 |
